# Significant individual change should be used as a lower bound for anchor based estimates of meaningful change on patient-reported outcome scores

**DOI:** 10.1007/s11136-024-03788-9

**Published:** 2024-09-28

**Authors:** John Devin Peipert, David Cella, Ron D. Hays

**Affiliations:** 1 Department of Medical Social Sciences, Northwestern University Feinberg School of Medicine, Chicago, USA; 2 UCLA Department of Medicine, Division of General Internal Medicine & Health Services Research, Los Angeles, USA; 3 Centre for Patient Reported Outcomes Research, Department of Applied Health Sciences, University of Birmingham, Edgbaston Birmingham, UK, Edgbaston, Birmingham, UK

**Keywords:** Interpretation, Important change, Meaningful within patient change

## Abstract

Interpretation of patient-reported outcome (PRO) scores has been supported by identifying score thresholds or ranges that indicate clinical importance. There has been a recent focus on the estimation of meaningful within patient change (MWPC). While much attention has been focused on anchor-based methods, some researchers prefer that a lower bound to these estimates should exceed a change score that could be observed due to measurement error alone as a safeguard against misclassifying individual patients as changed when they have not. The standard error of measurement (SEM) is often used as the lower bound of anchor estimates. Here, we argue that the SEM is not an the best lower bound for MWPCs. Instead, statistically significant individual change as calculated by the reliable change index (RCI) should be used as the lower bound. Our argument is based on two points. First, conceptually, the SEM does not provide specific enough information to serve as a lower bound for MWPCs, which should be based on the level of observed score change that is unlikely to be due to chance alone. Second, the SEM is not appropriate for direct application to observed scores, and requires a multiplier when examining observed change instead of true change. We conclude with recommendations for using the RCI with a thoughtful range of p-values in combination with anchor estimates.

## Introduction

Identifying score thresholds or ranges that indicate clinical importance has assisted in interpreting patient-reported outcome (PRO) scores [1–3]. The increasing use of PROs to guide clinical care and evaluate emerging treatments, including for regulatory decision-making, has increased the need for clearly interpretable PRO scores to indicate when a clinical action is required and to define treatment benefits and risks [4, 5]. This activity has renewed interest in which methods are most appropriate to identify PRO change score thresholds, though this has been an active area of research for decades. The recent focus has been on estimating meaningful change for patients to apply at the individual patient level [6, 7]. Methodological development in this area has been directed at identifying cut-points or ranges representing a meaningful improvement or worsening on the concept of interest. In turn, these cut points or ranges can be used to classify individual patients as treatment responders or failures [8]. The United States Food and Drug Administration (FDA) recommends this approach and has published guidance emphasizing estimating meaningful within-person change (MWPC) thresholds. These methods rely on anchor analyses, where an external variable (the anchor) with clinically meaningful and distinct groups is used to identify change thresholds on the PRO score [9]. Anchor-based methods are preferred over distribution-based methods because they include information directly relevant to patients or that is clinically meaningful. The FDA’s Patient-Focused Drug Development Draft Guidance: *Incorporating Clinical Outcome Assessments Into Endpoints for Regulatory Decision-Making*, notes that: “Distribution-based methods (e.g., effect sizes, certain proportions of the standard deviation and/or standard error of measurement) do not directly consider the patient voice, and as such, are insufficient to serve as the sole basis for identifying an [meaningful score difference]. Distribution-based methods can provide helpful information about measurement variability” [4]. Similarly, there has been an emphasis on identifying PRO change thresholds that can indicate to a clinician when an individual patient has improved or declined at a level that the patient finds meaningful and suggests a need to change treatment strategies [10].

Changes in individual PRO scores tend to be unreliable. This makes individual-level change scores more susceptible to occurring by chance alone, even when the patient has not changed [11, 12]. Measurement error is assumed to be distributed on both sides of true change such that it can generate false positives and false negatives. However, commonly used anchor methods may underestimate the threshold needed to indicate meaningful change [13]. This leads to the additional risk that individuals will be classified as changed when they have not by setting the threshold too low. For this reason, it may be useful to set a lower bound to anchor-based estimates of the MWPC, which would be considered only if they are larger than the measurement error in the PRO score [14, 15]. Requiring that the minimum or lower bound of the MWPC range exceed a change score that could be observed due to measurement error alone is a safeguard against misclassifying individual patients as changed when they have not.

One statistic used for the MWPC lower bound is the standard error of measurement (SEM, which can be thought of as, “the amount or spread in the measurement errors for a test.” [16] ^(p. 33)^ One aspect of the SEM’s appeal was that it appeared to capture the amount of error in a PRO score over which an observed PRO change should surpass to be counted as real change. There was some evidence that it aligned with anchor-based estimates of important change [17–19]. The SEM was seen, for example, as an alternative to criteria like the ½ standard deviation, which had been suggested as a reasonable lower bound or starting place for meaningful difference or change estimates [20]. In subsequent research, the SEM was cited as a lower bound to anchor-based meaningful change estimates [21, 22]. Moreover, using statistical indexes of individual patient change has been standard practices for decades in clinical psychology, and continues to be so today [23].

Though the SEM is an improvement over some distribution estimates previously used (e.g. a fraction of a standard deviation), two issues remain with using the SEM as a lower bound of MWPC. First, conceptually, the SEM does not provide specific enough information to serve as a lower bound for MWPCs, which should be based on the level of observed score change that is unlikely to be due to chance alone. Second, as demonstrated by Christensen and Mendoza, the SEM is not appropriate for direct application to observed scores, and requires a multiplier when examining observed change instead of true change [24]. We argue that the SEM is not the best lower bound for MWPCs. Instead, the statistically significant individual change as calculated by the reliable change index (RCI) using thoughtful statistical thresholds should be used as the lower bound. Our argument is based on two points. We conclude with recommendations for using the RCI and anchor estimates of the MWPC. By identifying the appropriateness of the RCI as a lower bound to MWPC thresholds instead of the SEM, we help protect against a known pitfall in over-reliance on anchor estimates at a time when their use is increasing. Specifically, this work responds to previous studies that address the problem of when anchor estimates of meaningful change fall below the threshold of change that is detectable due to measurement error [15, 25].

## Statistical indexes of individual change

Multiple statistical approaches to index individual change deal in some way with measurement error. A typical formula to estimate the SEM is: ${SD}_{1}\sqrt{1-reliability}$, where SD_1_ is the standard deviation of the score at baseline, and the reliability is typically internal consistency or test–retest reliability [26].

The RCI [27], or the derivative likely change index (LCI) [28], provide a test of statistically significant change at the individual patient level. The current form of the RCI was introduced by Jacobson and Truax in 1991 [28] and is defined as ${(X}_{2}-{X}_{1})/(\sqrt{2}*SEM)$, where X_1_ is a particular individual patient’s PRO score at baseline, X_2_ is the same individual’s PRO score at a follow-up timepoint, and SEM is defined as above. When an individual’s RCI value is ≥ 1.96, their change on the PRO is statistically significant at p < 0.05, since 1.96 is the critical value for a p-value of 0.05. Since the RCI at p < 0.05 tends to result in large thresholds for statistical significance, more inclusive thresholds have been recommended when a researcher does not require the certainty of a p-value of < 0.05 [28]. The LCI is equivalent to the RCI but was introduced to emphasize that the 1.96 critical value for the RCI at p < 0.05 can be relaxed to more permissive p-values [e.g., 1.65 for p = 0.10, 0.994 for p = 0.32 (~1 standard deviation from 0 on standard normal distribution)]. The RCI can be transformed to a coefficient of repeatability using the following equation, which represents the amount of change on the PRO needed to reach statistical significance: $\sqrt{2}*SEM$ multiplied by the critical value associated with the p-value of interest (e.g., 1.96; 1.65; 0.994). These formulae are summarized in Table 1. The SEM and the RCI have been proposed as thresholds to determine individual PRO change, since relying on anchor estimates based on group-level averages may generate small values that can occur by chance [14, 28, 29]. In other words, an anchor estimate of meaningful change may fall below the score change that is detectable due to measurement error. This problem with anchor estimates has been previously pointed out [25]. Kemmler, et al. proposed a solution to this problem by increasing all anchor estimates that fall below 1 standard deviation to this value, which always exceeded the threshold of statistically significant change [25]. Terwee, et al. responded to this solution by recommending using very high reliability PROs so that the smallest detectable change is not larger than the anchor estimate of meaningful change [15]. Below, we describe a novel and flexible solution to this issue that employs the RCI as a lower bound but acknowledges that the thresholds set by the RCI using a p-value of < 0.05 might be too high for some applications.

**Table 1:** Formula for Calculating Significant Individual Change

Statistic	Formula
Standard error of measurement (SEM)	${SD}_{1}\sqrt{1-reliability}$
Reliable/Likely Change Index^a^ (RCI/LCI)	${(X}_{2}-{X}_{1})/(\sqrt{2}*SEM)$
Coefficient of Repeatability	$Critical value*\sqrt{2}*SEM$

^a^The RCI and LCI have the same formula. The LCI uses a p-value of > 0.05 to determine significance

## The RCI (LCI) has advantages over the SEM as an MWPC lower bound

The RCI has advantages over the SEM in setting a lower bound to an MWPC range. First, the SEM estimates the measurement error in a PRO score, which is helpful to know, but does not indicate how likely an observed PRO change score threshold is to occur by chance alone. If we want to have reasonable certainty that changes in scores exceeding anchor-based estimates of MWPC are not observed merely due to chance, we need a statistic like the RCI. The difference in suggested thresholds for individual change between the SEM and RCI/LCI is material, as is evident from their formula. For example, Hays et al. calculated the SEM and RCI in SF-36 scales (reliabilities ranging from 0.77 to 0.94) in a study of 54 patients visiting UCLA’s Center for East–West Medicine and found the RCI required observed change scores to be 2–58% larger than the SEM to surpass the threshold for statistically significant change using p < 0.05 [30]. In a simulation study, Terluin showed that when the prior probability of true change was 50% and measure reliability was 0.90, the RCI at p-values of 0.50 (equivalent to 0.95 SEM), 0.32 (equivalent to 1.41 SEM), and 0.05 (equivalent to 2.77 SEM) were associated with 80%, 87%, and 97% probabilities of true change > 0 on a PRO score, but these probabilities dropped to 38%, 48%, and 77%, when prior probability of true change was 10% and the reliability was 0.70 [31]. Noting that the RCI with a p-value of 0.50 is nearly equivalent to the SEM, this research shows that, depending on reliability, SEM-based criteria for individual change may result in unacceptably low probabilities of real change and unacceptably high probabilities that observed change scores are due to chance alone.

Our second argument against using the SEM as an MWPC lower bound taps into a problem pointed out nearly 40 years ago. The original version of the RCI, published in 1984, differed from the revised version cited above in that the denominator was only the SEM and did not include the $\sqrt{2}$ term [32]. In 1986, Christensen and Mendoza pointed out that since the SEM is the spread of *observed* scores around the true score for a single patient, the RCI, in its original form, was an index of change from a *true* score at baseline [24]. However, as the basis of CTT is that observed scores are the combination of true score and error [33], the assumption that the X_1_ (baseline) score from the RCI formula represents a *true* score without measurement error is untenable for the vast majority of applications with PROs that use CTT-based scoring. Instead, the RCI needed to index changes from an observed score at baseline. Christensen and Mendoza then argued that the SEM (SEM at baseline) in the denominator of the RCI should be replaced with the standard error of the difference between X_1_ and X_2_, resulting in a statistic that was approximately $\sqrt{2}$ of the RCI in its original formulation [24]. This led to inclusion of the $\sqrt{2}$ term in the denominator of the revised RCI [27]. Christensen and Mendoza’s lesson for the original RCI also applies to setting an MWPC lower bound. The MWPC lower bound should consider how error impacts change from an observed score at one time to an observed change score at a second time, since that will be the context in which MWPCs are also used. Reliance on the SEM without adjustment by $\sqrt{2}$ may lead to MWPC lower bounds that are too low.

## Conclusions and recommendations

Here, we have argued that the lower bound of an MWPC range based on anchors should be the RCI instead of the SEM due to their advantages of providing the probability that any PRO change would be observed by chance alone and that they consider change between two observed scores, instead of change relative to a true score. The differences between the SEM and RCI or LCI may seem academic, but use of one over the other will have noticeable ramifications for the MWPC lower bound that is set. The RCI statistics can be considered useful approaches to put the SEM into better practice for understanding change in PRO scores.

When computing the RCI (or LCI), it is very important to decide whether to use the conventional, originally proposed p-value of 0.05, or to use a larger p-value. Setting the p-value at 0.05, or even 0.10, will provide a high degree of certainty that the observed change is real. However, it may also misclassify many people who have changed as unchanged. Setting the p-value lower (e.g., at 0.32) may do a better job of accurate classification overall regarding how much change patients feel is meaningful, but could increase the number of “false positive” classifications [28]. In some cases, there may be more tolerance for the likelihood that an observed score is due to chance alone, while there may be calls for a more conservative limit in others. Moreover, the measure’s reliability should be considered; higher reliability should grant researchers more leverage with higher p-values. Measures with low reliability should require very high standards for interpretation at the individual level or should not be used with individuals at all.

The choice of a p-value should be based on the PRO’s reliability and the researcher’s tolerance for chance observation in the particular application. We have summarized initial guidance on selecting an RCI p-value in Table 2. The table expresses the individual change in terms of the coefficient of repeatability since it calculates thresholds on the PRO scale for significant individual change. We suggest three potential p-values: p < 0.05, p < 0.32, and p < 0.50. p < 0.05 is the conventional value. p < 0.32 was suggested by Donaldson et al. because it reflects the point of 1 standard deviation from the mean on a probability function where diminishing probabilities as reflected on a curve flatten out [34]. p < 0.50 reflects an equal probability that the observed change score is due to chance alone, and is very close to 1 SEM. This may be acceptable for maximizing accurate classification with highly reliable tests. In a previous analysis, thresholds generated by the RCI with a p-value of < 0.05 tended to be higher than anchor estimates. In contrast, higher p-values up to p < 0.50 were similar or slightly lower than anchor estimates [28]. As noted above, no one-size-fits-all recommendation can be given for the selection of the p-value. The advantage of using an RCI/LCI statistic as a lower bound to anchor estimates is its flexibility; a researcher can select a p-value-based threshold under which the likelihood of chance observation is unacceptable. In terms of procedure, a researcher can estimate the MWPC using anchor methods as described above, then select a coefficient of repeatability using guidance from Table 2 to serve as a lower bound to the anchor estimated MWPC range, omitting any anchor estimates lower in magnitude than the selected coefficient of repeatability.

**Table 2:** Guidance for Selecting a Coefficient of Repeatability (CR) P-value

Reliability		Level of Tolerance for Chance Observations^a^		
		Low	Medium	High
Low				
	~ 0.70	None	p < 0.32	p < 0.32–0.50
	~ 0.80	p < 0.05 to < 0.32	p < 0.32	p < 0.32–0.50
	~ 0.90	p < 0.05 to < 0.32	p < 0.32	p < 0.32–0.50
High				

~Represents approximate levels of reliability

^a^“Chance observations” refers to varying levels of likelihood that the change score magnitude evaluated for meaningfulness would be observed by chance alone

We note that, in some cases, individual patients may be incorrectly coded as not having changed when they have due to measurement error, i.e., the error generated a false negative. The chances of this happening may be exacerbated if the RCI, with p-value of < 0.05, is used as a lower bound to the MWPC, as this statistic tends to have high change thresholds. In cases where it is suspected that too many people who have changed would be incorrectly coded as not having changed, we recommend use of the LCI. This may be the case in larger studies where the results of classifying individual patients as having changed are then aggregated into groups for analysis (e.g., responder analysis between treatment arms of a clinical trial); indeed, the effects of measurement error on misclassification in terms of false positives and negatives are likely to balance out in the aggregate. However, in cases where individual change is the unit of interpretation, such as earlier phase clinical trials and clinical practice, researchers may want to select a more stringent basis for the change threshold. Finally, a limitation of the current work is the lack of applicability to PROs using item-response theory scores, which have different ways of handling measurement error, and entail different approaches to calculating significant individual change.[35] Future research in lower-bound setting for measures based on item-response theory should expand on the research presented here.
